# Higher Tumor Mutation Burden and Higher PD-L1 Activity Predicts the Efficacy of Immune Checkpoint Inhibitor Treatment in a Patient With Four Lung Cancers. A Case Report

**DOI:** 10.3389/fonc.2020.00689

**Published:** 2020-06-02

**Authors:** Katsuo Usuda, Yo Niida, Shun Iwai, Aika Funasaki, Atsushi Sekimura, Nozomu Motono, Sohsuke Yamada, Hidetaka Uramoto

**Affiliations:** ^1^Department of Thoracic Surgery, Kanazawa Medical University, Ishikawa, Japan; ^2^Center for Clinical Genomics, Kanazawa Medical University, Ishikawa, Japan; ^3^Division of Genomic Medicine, Kanazawa Medical University, Ishikawa, Japan; ^4^Department of Pathology and Laboratory Medicine, Kanazawa Medical University, Ishikawa, Japan

**Keywords:** lung cancer, immune checkpoint inhibitor (ICI), programmed death ligand 1 (PD-L1), tumor mutation burden (TMB), microsatellite instability (MSI)

## Abstract

We experienced a patient who had four lung cancers with different pathological features, with the most advanced being diagnosed as pStage IIA. A month after the resection, the original lung cancer had metastasized to the lung and to the liver. Of the original lung cancers that were resected, the biggest adenocarcinoma of S3 showed 50 × 31 × 17 mm (invasion 50 mm) and pT2bN0M0 (pStage IIA) with epidermal growth factor receptor (EGFR) mutation (–) and anaplastic lymphoma kinase (ALK) translocation (–), but expression of programmed death ligand 1 (PD-L1) (+) tumor proportion score (TPS) 80%. The pleomorphic carcinoma showed 23 × 20 × 17 mm (invasion 23 mm) and pT1cN0M0 (pStage Ic) with EGFR (–), ALK (–), PD-L1 (+), TPS 95%. Tumor mutation burden (TMB), microsatellite instability (MSI), and structural chromosome aberration analysis by DNA microarray were performed. One hundred somatic mutations in the adenocarcinoma and 75 somatic mutations in the pleomorphic carcinoma were identified, which showed an extremely high mutation rate, although only 16 somatic mutations were common between the two cancers. Chromosomal structural aberrations differed greatly between the two cancers, but common aberrations were found in chromosomes 8 and 10 and partially common aberration in chromosomes 4, 14, 17, and X. These results indicated that each lung cancer originated from a common ancestor clone and developed on an individual molecular evolution. The patient received a single injection of pembrolizumab and 13 injections of atezolizumab. Immune checkpoint inhibitor treatment made metastatic pulmonary and liver lesions reduce in size and show as Partial response (PR). Multiple lung cancers with high PD-L1 activity tend to be TMB-high, reflecting rapid molecular evolution and relevance to the patient's response to immune checkpoint inhibitors. Genomic examination could help determine what had happened in multiple cancers on progression and provide useful data to patient treatment. Each lung cancer originated from a common ancestor clone and developed on an individual molecular evolution.

## Introduction

Clinically valuable prognostic and predictive biomarkers that can reliably determine the patients most likely to benefit from adjuvant chemotherapy are still in the developmental stages. The recent progress of antibodies that target the programmed death 1 (PD-1) as well as programmed death ligand 1 (PD-L1) pathways plays critical in the management of advanced non–small cell lung cancer (NSCLC). Similar to the PD-L1 expression, tumor mutation burden (TMB) and microsatellite instability (MSI) are being used for the first time as predictive biomarkers for identifying patients likely to positively react to immune checkpoint inhibitors (1–5). However, the overlap between the PD-L1, TMB, and MSI differs among cancer types, and only 0.6% of cases were positive for all the three markers in malignant tumors (3).

We herein report a patient with four lung cancers who had a remarkable response to immune checkpoint inhibitors for NSCLC. The TMBs of the lung cancers were identified to be 105.9 mutations per megabase (mut/Mbp) in adenocarcinoma and 79.4 mut/Mbp in pleomorphic carcinoma by next-generation sequencing analysis of cancer gene panel test, SureSelect NCC Oncopanel (Agilent) National Cancer Center Hospital in Japan, Tokyo, Japan.

## Case Presentation

**Figure 1:**
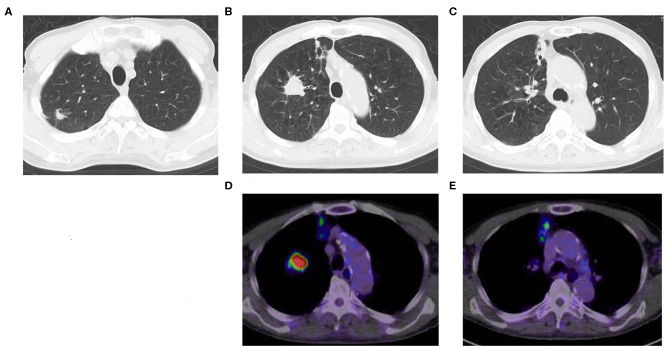
Chest CT shows multiple lung cancers. **(A)** Adenocarcinoma, **(B)** pleomorphic carcinoma, **(C)** adenocarcinoma, **(D)** positron emission tomography computed tomography (PET-CT) of pleomorphic carcinoma **(B)**, **(E)** PET-CT of adenocarcinoma **(C)**.

A CT scan showed that an 87-year-old man had three nodules in his right upper lobe of the lung (Figure 1). These nodules were highly suspected to be cancerous (cT2bN0M0, cStage IIA), and he underwent a right upper lobectomy and lymphadenectomy of ND1 for lung cancer under a complete video-assisted thoracoscopic surgery procedure. A pathological examination revealed that out of the four lung cancers one was a pleomorphic carcinoma, and three were adenocarcinomas. The biggest adenocarcinoma of S3 showed 50 × 31 × 17 mm (invasion 50 mm) and pT2bN0M0 (pStage IIA) with epidermal growth factor receptor in real-time polymerase chain reaction (PCR) (EGFR) (–), anaplastic lymphoma kinase (ALK) (–), and expression of PD-L1 (+) tumor proportion score (TPS) 80%. The pleomorphic carcinoma showed 23 × 20 × 17 mm (invasion 23 mm) and pT1cN0M0 (pStage Ic) with EGFR (–), ALK (–), and PD-L1 (+) TPS 95%. One month after resection, there were multiple metastases to lung and liver that had developed quickly. The liver metastasis was diagnosed pathologically as a metastasis of the pleomorphic carcinoma by percutaneous liver biopsy.

**Figure 2:**
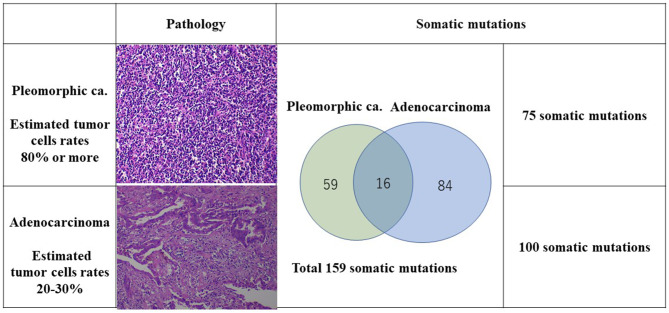
Relationship between pathology and somatic mutations.

**Figure 3:**
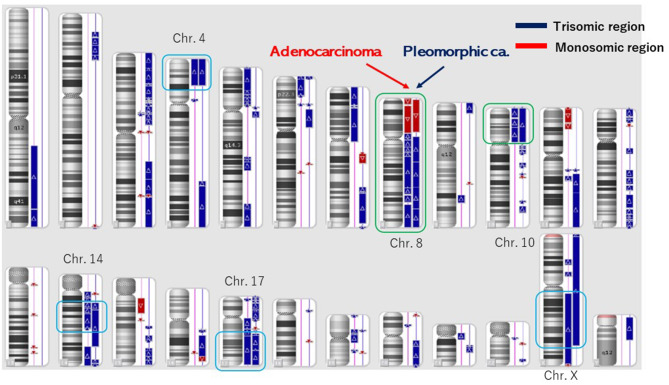
Structural chromosome aberration analysis by OncoScan CNV. Common chromosomal aberrations were found in chromosomes 8 and 10, and the process that piled up independent chromosomal aberrations was inquired of these tumors having a common origin.

The TMB and MSI were analyzed under SureSelect NCC Oncopanel (Agilent) and Bethesda panel assay in the patient's lung cancer samples. Also, OncoScan CNV (Affymetrix) Thermo Fisher Scientific, Tokyo, Japan was performed to analyze chromosomal aberrations (Figures 2, 3). A pleomorphic carcinoma had a uniform carcinoma which cancer cells had occupied more than 80% of the central tumor area (Figure 2). The biggest adenocarcinoma had a un-uniform adenocarcinoma with stroma and lymphocytic infiltration which cancer cells had occupied 20 to 30% of the central tumor area. The second adenocarcinoma showed an arrangement of islands. The third adenocarcinoma showed adenocarcinoma *in situ* and normal tissue. The second and third adenocarcinomas were not trimmed for carcinoma tissue for DNA analysis. Finally, DNA was extracted from the formalin-fixed paraffin-embedded tissues of the pleomorphic carcinoma and the biggest adenocarcinoma. DNA from the normal tissue was used as the normal control.

The number of target bases of NCC Oncopanel was 944,153 bp (0.944 Mb). Therefore, when TMB was defined as the total number of somatic mutations per 1-Mb read, the TMB was identified to be 79.4 mut/Mbp in the pleomorphic carcinoma and 105.9 mut/Mbp in the adenocarcinoma. Finally, 75 somatic mutations were identified in the pleomorphic carcinoma and 100 somatic mutations in the adenocarcinoma, which showed an extremely high hypermutation rate, although only 16 somatic mutations were common between the two cancers. There were no instabilities of the microsatellite in both the adenocarcinoma and the pleomorphic carcinoma, and it was judged as microsatellite stable (MSS). The adenocarcinoma had a driver mutation of L858R of EGFR assumed to be homozygous; variant allele frequency is 0.278, with tumor content of the sample being 20 to 30%, and other somatic mutations' allele frequencies were divided into two groups, with average 0.124 (*n* = 58) and 0.308 (*n* = 42), which seemed to represent heterozygous and homozygous mutations, respectively. These results indicate that this adenocarcinoma has uniform genetic characteristics. On the other hand, although the pleomorphic carcinoma presented a uniform pathologic finding, no specific driver mutation was found, and average allele frequencies of somatic mutations are divided to 0.98 (*n* = 1), 0.28 (*n* = 42), and 0.121 (*n* = 32). Because tumor content of the sample is ~80%, the former two represent homozygous and heterozygous mutations, respectively, but the last one indicates that only a part of the tumor has these mutations. Therefore, it is assumed that this pleomorphic carcinoma is heterogeneous at the molecular level. Structural chromosome aberration analysis by DNA microarray showed great difference between two tumors, but also common chromosomal aberration in chromosomes 8 and 10 and partially common chromosomal aberration in chromosomes 4, 14, 17, and X (Figure 3). Higher TMB and higher PD-L1 activity predicted a positive response to the immune checkpoint inhibitor in this patient.

**Figure 4:**
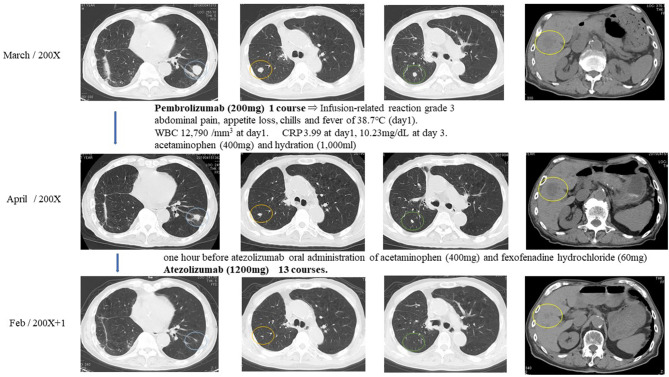
Response evaluation was shown as PR after intravenous injection of immune checkpoint inhibitors.

One hour after pembrolizumab (200 mg) had been given to the patient intravenously, he had right abdominal pain, appetite loss, chills, and a fever of 38.7°C. White blood cell count increased to 12,790/mm^3^ on day 1 and decreased until it finally stabilized on day 2. C-reactive protein was 3.99 on day 1 and increased to 10.23 mg/dL by day 3 until finally stabilizing. The episode was judged as an infusion-related reaction of grade 3. The patient received acetaminophen (400 mg) and hydration (1,000 mL), which led to the amelioration of symptoms. One month after pembrolizumab injection, multiple metastatic pulmonary and liver tumors were reduced in size and judged as PR (Figure 4).

Because of the pembrolizumab infusion-related reaction, pembrolizumab was discontinued, and atezolizumab was selected for the immune checkpoint inhibitor treatment. The patient received acetaminophen (400 mg) and fexofenadine hydrochloride (60 mg) 1 h before atezolizumab injection to prevent infusion-related reactions. The patient tolerated atezolizumab (1,200 mg) without any subsequent infusion-related reactions. Atezolizumab was given to the patient intravenously every 3 weeks for 13 courses. Multiple metastatic pulmonary lesions were reduced in size and almost disappeared, and multiple metastatic liver tumors were reduced in size. Overall response evaluation of immune checkpoint inhibitors at 12 months after the treatment was judged as PR (Figure 4).

## Discussion

Programmed death 1 (PD-1) and programmed death ligand 1 (PD-L1) inhibitors in the treatment of advanced NSCLC are currently available and have demonstrated antitumor activity (6–8). PD-1 and PD-L1 inhibitors significantly improved the overall survival (OS), progression-free survival, and objective response rate in patients with advanced NSCLC with PD-L1 expression. Furthermore, a high PD-L1 expression was likely to be associated with increased benefits.

The TMB is defined as the total number of somatic mutations of the genomic coding area and associated with the emergence of neoantigens that trigger antitumor immunity (9). The TMB was recently confirmed to be a biomarker of the efficacy of PD-1 and PD-L1 inhibitors (1, 5). Although objective cutoff points for the TMB are not universally established, the cutoff points have been set at around 10 mut/Mbp in previous studies. Therefore, our case, whose TMBs were 105.9 mut/Mbp in adenocarcinoma and 79.4 mut/Mbp in pleomorphic carcinoma, was defined as TMB-high. High nonsynonymous TMB (>8 mut/Mb) was prognostic for favorable outcomes in patients with resected NSCLC (10). High nonsynonymous TMB was shown to have a better prognosis in patients with resected NSCLC (10). Multivariate analysis demonstrated clonal mutation burden as a promising independent prognostic factor for OS in SCLC patients (11). High TMB (> 21 mut/Mb) was a good prognostic factor in OS (21.7 vs. 10.4 months, *P* = 0.012) (12). Multivariate analysis presented that high TMB was an independent prognostic factor (12).

However, other chromosomal aberrations were greatly different between the pleomorphic carcinoma and the adenocarcinoma, and the process of independent chromosomal aberration was assumed, although these tumors had a common origin. The pleomorphic carcinoma presented a uniform pathologic finding, but suspected to have various subclones genomically. Although the pleomorphic carcinoma did not have a specific driver mutation, it could become a good target of immune checkpoint inhibitors reflecting high somatic mutation rate. The adenocarcinoma would have a driver mutation of L858R of EGFR in homozygous and be relatively uniform genomically in NCC Oncopanel. There is an apparent discrepancy between negative EGFR (Cycleave methods) expression in real-time PCR and a putative EGFR driver mutation (L858R) in NCC Oncopanel in the adenocarcinoma. The discrepancy was thought to be based on the methods of EGFR examinations. In our experience, pleomorphic carcinomas rarely express an EGFR expression. In this case, the pleomorphic carcinoma with no specific driver mutation of EGFR had metastases to the liver and the lungs. Epidermal growth factor receptor–tyrosine kinase inhibitor (EGFR-TKI) was not used because of the negative expression of EGFR. When the adenocarcinoma relapsed, EGFR-TKI was used based on the NCC Oncopanel.

Microsatellite instability is also considered an independent predictive biomarker of immune checkpoint inhibitors (2, 3). Microsatellite instability is the situation of genetic hypermutability and represents the phenotypic results of mismatch repair (MMR) deficiency. Cancers with instability at two or more of these loci are defined as MSI-high (MSI-H), whereas those with instability at a single locus are defined as MSI-low (MSI-L), and those with no instability at any of these loci are defined as MSS. Whereas the examination for MSI is MSS, the frequent occurrence of the mutation would not mean the failure of the MMR mechanism. In addition to the PD-L1 expression, MSI would be a different biomarker from TMB, and TMB and MSI are considered independent predictive biomarkers for selecting patients likely to benefit from immune checkpoint inhibitor treatment.

The overall rates of PD-L1 positivity, MSI-H, and TMB-high of cancers were 3.0, 7.7, and 25.4%, respectively (3). Only 0.6% of the cancer cases were positive for all three markers (3). The overlap rates between PD-L1, TMB, and MSI were reportedly low in NSCLC (3): TMB-high and MSI-H were found in 0.5%, TMB-high and PD-L1 positivity in 7.7%, MSI-H and PD-L1 positivity in 0.4%, and positivity in all three markers in 0.3% of NSCLC cases (3). This reported case was classified as TMB-high, PD-L1 positivity, and MSS. These data present several different mechanisms in this case. This discrepancy may be attributed to other biomarkers associated with the efficacy of PD-l and PD-L1 inhibitors. Programmed death ligand 1 expressions and TMB were independent variables, and a composite of PD-L1 plus TMB further supported the efficacy of immune checkpoint inhibitor treatment (13). Higher PD-L1 TPS scores as well as higher TMB of both lung cancers indicated strongly immune checkpoint inhibitors for the therapy. Higher PD-L1 TPS scores of this different lung cancers showed to have a common origin, which was supported by the 16 common somatic mutations. In these lung cancers, common chromosomal aberrations were found in chromosomes 8 and 10, which showed two different lung cancers originated from the same origin and then differentiated independently. These lung cancers had the same gene abnormality of PD-L1 positivity, TMB-high, MSS, and common chromosomal aberration, which presented the same origin instead of different cell types.

The patient had multiple lung cancers of different histologic types, which showed a postoperative immediate relapse. The lung cancer was TMB-high, so the immune checkpoint inhibitor proved to be successful in treating the relapse. The cancer genome analysis showed the lung cancers had different genetic backgrounds. One of which was an adenocarcinoma, and the other, a pleomorphic carcinoma, but both would have developed from a common ancestor. They share some similar molecular evolution that differs from their common ancestor but eventually followed independent evolutionary lines. We think this was a rare case that had simultaneous examinations of gene panel test and microarrays. The speed of the mutation in the gene was high, so it contributed to the simultaneous cancer developments, and therefore TMB was super high, and the immunity checkpoint inhibitors were good indicators. Whereas the chromosomal aberration looks remarkable, but it is only MSS. There is no homologous recombination deficiency, showing no mutation of the DNA MMR gene.

Infusion-related reactions in immune checkpoint inhibitor treatment occur in ~3% of patients, and recognition of infusion-related reactions induced by pembrolizumab is an important aspect of its usage (14, 15). Corticosteroid, antihistamine, and antipyretic analgesics were reported to be useful for prevention of infusion-related reactions (14).

One limitation of this study was the lack of genomic data of metastatic lesions. There was not enough carcinoma tissue of the metastatic liver tumor to extract DNA. When two or more lung cancers are detected or other malignancies are detected at the condition of PD-L1 (+), there will be the possibility of a common genomic abnormality of TMB-high. One of the strengths of this study was the existence of the pathologically and genomically sufficient data of multiple simultaneous lung cancers and the outcome for immune checkpoint inhibitors.

## Conclusion

Multiple lung cancers with high PD-L1 activity tended to be TMB-high reflecting rapid molecular evolution and relevance to the patient's response to immune checkpoint inhibitors. Genomic examination could help determine what will happen in multiple cancers on progression and provide useful data for patient treatment. Each lung cancer originated from a common ancestor clone and developed on an individual molecular evolution.

## Ethics Statement

Written informed consent was obtained from the patient for the publication of any potentially identifiable images or data included in this article.

## Author Contributions

KU performed the research, wrote the paper. SI, AF, AS, and NM were performed therapy to a patient. YN contributed to analyze the genetic status of this patient. SY performed pathological examination of lung cancers. HU contributed to supervision of this study and revision of the manuscript.

## Conflict of Interest

The authors declare that the research was conducted in the absence of any commercial or financial relationships that could be construed as a potential conflict of interest.
